# Black population’s health in nursing training: students’ knowledge

**DOI:** 10.1590/0034-7167-2024-0330

**Published:** 2025-06-20

**Authors:** Gabriela Matos Rodrigues, Beatriz de Souza Lima, Eduardo Sodré de Souza, Hugo Fernandes, Suiane Costa Ferreira, Flávia Saraiva Leão Fernandes

**Affiliations:** I Universidade Federal de São Paulo. São Paulo, São Paulo, Brazil; II Faculdade Sírio-Libanês. São Paulo, São Paulo, Brazil; III Universidade Estadual de Campinas. Campinas, São Paulo, Brazil; IV Universidade do Estado da Bahia. Salvador, Bahia, Brazil

**Keywords:** Education, Nursing, Teaching, Race Relations, Racism, Black People, Educación en Enfermería, Enseñanza, Relaciones Raciales, Racismo, Población Negra

## Abstract

**Objectives:**

to analyze the knowledge of students and recent graduates of a nursing course about the black population’s health.

**Methods:**

an exploratory and cross-sectional study with nursing students and recent graduates from a public university in southeastern Brazil. Data were collected online with 63 participants. The Kruskal-Wallis test was performed for statistical analysis.

**Results:**

there was little knowledge about the black population’s health and the Brazilian National Policy for Comprehensive Black Population’s Health. The approach to this topic in the nursing course is rare and mainly carried out in collective health curricular units, from a socio-historical perspective.

**Conclusions:**

students’ low knowledge reveals the need for a greater approach to the black population’s health in the nursing course, with a view to training centered on ethnic-racial relations and anti-racist practices.

## RESUMEN

**Objetivos:**

analizar el conocimiento de estudiantes y recién egresados de un curso de enfermería sobre la salud de la población negra.

**Métodos:**

estudio exploratorio y transversal, con estudiantes de enfermería y recién graduados de una universidad pública del sudeste de Brasil. Los datos se recopilaron en línea de 63 participantes. Para el análisis estadístico se realizó la prueba de Kruskal-Wallis.

**Resultados:**

se evidenció bajo conocimiento sobre la salud de la población negra y sobre la Política Nacional Integral de Salud para la Población Negra. El abordaje del tema en los cursos de enfermería es poco común y se realiza principalmente en unidades curriculares de salud colectiva, desde una perspectiva sociohistórica.

**Conclusiones:**

el bajo conocimiento de los estudiantes revela la necesidad de un mayor abordaje de la salud de la población negra en la carrera de enfermería, con miras a una formación centrada en las relaciones étnico-raciales y las prácticas antirracistas.

## INTRODUCTION

Scientific evidence on health education reveals the huge gap that exists in the debate about racism and the black population’s health, which reveals the urgency of rethinking health education in order to reduce racial inequities and meet the principles of universality, comprehensiveness and equity in the Brazilian Healthcare system (In Portuguese, *Sistema Único de Saúde* - SUS)^(1)^.

Although the SUS legally guarantees universal and equal access to healthcare, black and indigenous populations face daily barriers to accessing healthcare, which are reflected in their health, illness and death outcomes. These barriers are imposed by institutional racism, which is evidenced in the comparative analysis of epidemiological data disaggregated by race/color, such as early deaths, high maternal and infant mortality rates, higher prevalence of infectious and chronic diseases, and higher rates of violence^(2, 3)^.

Evidence of negative attitudes of healthcare professionals when providing care to the black population reflects the interpersonal dimension of institutional racism in services^(2)^. Such attitudes, manifested through incompetence, recklessness and negligence, contribute to health inequities among this group, while also announcing the need to address the issue in health courses^(4)^.

The nursing field has a privileged position in healthcare systems and, therefore, has a great capacity to produce changes that contribute to dismantling institutional racism. However, the low level of knowledge and studies on this population’s health calls for further research in this field. Therefore, the discussion on the impacts of racism on the social determination of disease and actions to address these inequalities should be included in future nursing professionals’ teaching-learning process, in order to promote health equity in Brazil and meet the Brazilian National Policy for Comprehensive Black Population’s Health (In Portuguese, *Política Nacional de Saúde Integral da População Negra* - PNSIPN) guidelines^(5, 6)^.

The result of intense participation by the black movement and organized black women, PNSIPN advocates the production of content, training and continuing education for health workers. Its general guideline is the “inclusion of topics of racism and black population’s health in health workers’ training and continuing education processes and in the exercise of social control in health”^(6)^. Thus, the role of public universities stands out, due to their ability to disseminate knowledge and increase its relevance in the means of learning and academic training, making viable the transformations that can be achieved in the reorganization of healthcare through the adoption of globalizing and inclusive institutional actions^(7)^.

In this context, the *Universidade Federal de São Paulo* (UNIFESP) has incorporated PNSIPN guidelines into its institutional documents. In its Institutional Development Plan 2021-2025^(8)^, it proposes to coordinate with policies to reduce ethnic-racial inequalities, in addition to renewing pedagogical projects and academic practices to combat racism and consolidating an institutional policy on the subject. In relation to this last proposition, the university relies on the Carolina Maria de Jesus Policy for the Promotion of Ethnic-Racial Equity and Equality, Prevention and Combat of Racism, which has as one of its axes “initial and continuing education in education for ethnic-racial relations”.

Given the institutional commitment taken over by UNIFESP and the importance of training future professionals to combat institutional racism in the SUS, it is argued that the analysis of knowledge about the black population’s health offered in nursing courses can contribute to identifying and filling existing gaps. Thus, this study had the following question: what is the knowledge of nursing students regarding the black population’s health?

## OBJECTIVES

To analyze the knowledge of students and recent graduates of a nursing course about the black population’s health.

## METHODS

### Ethical aspects

The research complied with the standards of Resolution 466/2012, and was submitted to the UNIFESP Research Ethics Committee. It was also approved by the nursing course committee. The Informed Consent Form (ICF) was submitted online and received the consent of those who voluntarily agreed to participate in the research.

### Study design, period and site

This is exploratory-descriptive research with a cross-sectional design, guided by the STrengthening the Reporting of OBservational studies in Epidemiology (STROBE) tool. Data were collected between March and April 2023 at the UNIFESP *Escola Paulista de Enfermagem.* The bachelor’s degree in nursing is a full-time, annual program, with a four-year completion period.

### Population or sample; inclusion and exclusion criteria

The study population consisted of nursing students and recent graduates from UNIFESP. The sample was obtained in a non-probabilistic manner, for convenience, by sending an online invitation to participate in the research to all people enrolled in the four years of the nursing course in March 2023 (288) and to all people who graduated at the end of 2022 (79), totaling 367 people.

Students enrolled in a nursing course or having graduated from the course less than one year ago at the time of data collection were included. Students who had suspended enrollment at the time of data collection or taking more than 60 days to respond to the instrument were excluded. The sample consisted of 63 participants, which corresponds to 17.2% of the total number of people invited to participate in the research.

### Study protocol

The invitation to participate in the research with the link to the self-administered virtual questionnaire was disseminated via the course’s academic secretariat to the electronic addresses of the four years of the undergraduate course and the class of graduates from the year prior to the data collection period. The virtual questionnaire was structured in the Research Electronic

Data Capture (REDCap) system, containing the ICF and the research instrument developed by the research team, for exclusive use in this study. The instrument consisted of closed-ended questions and two open-ended questions, divided into three parts: 1 - sociocultural profile; 2 - knowledge about the black population’s health and PNSIPN; and 3 - approach to the topic in the nursing course. To analyze knowledge, questions were developed with answers on a 5-point Likert scale (1 - totally disagree to 5 - totally agree), making it possible to analyze the level of agreement with statements about the black population’s health.

A pilot test was conducted with a group of five students using the same method that would be used with the other participants. The objective was to assess comprehensibility, objectivity, understanding, format, ease of completion, among others. The changes were based on feedback from students. The responses from the pilot test were not included in the final sample. Data collection was carried out after the project was approved by the Research Ethics Committee and adaptations from the pilot test were made.

### Analysis of results, and statistics

The Jamovi software was used for data analysis. In descriptive analysis of knowledge about the black population’s health, absolute and relative frequencies were calculated. In the association analysis, the year of graduation was used as the main variable. For comparison between groups (1^st^, 2^nd^, 3^rd^, 4^th^ years and recent graduates), the Kruskal-Wallis test was used, followed by the Dwass-Steel-Critchlow-Fligner post hoc test, which compares median/means of all pairs of groups and controls the error rate simultaneously for all contrasts. A significance level of 0.05 was considered for the interpretation of results.

## RESULTS

Sixty-three people participated in the study, nine in the first year (14.3%), 18 in the second year (28.6%), 12 in the third year (19%), 14 in the fourth year (22.2%), and ten recent graduates (15.9%). The sample was predominantly composed of women who were cisgender (90.5%), heterosexual (61.9%), without disabilities (96.8%), from the southeast (92%), belonging to the age group of 20 to 25 years (69.8%), whose average was 25 years ± 6.65. Concerning the race/color item, 68.3% identified themselves as white, and 23.8% identified themselves as black, of which 12.7% were brown and 11.1% were black. The smallest part was made up of yellow people (7.9%), and there were no participants who declared themselves indigenous.

The majority lived in the city of São Paulo (87.3%), with their parents (66.7%) and did not participate in any government income transfer program (84.1%), with an average income between three and five minimum wages (42.9%). As for education, 50.8% attended high school in a private school, and the majority were studying for their first degree (82.5%). Approximately 80.9% reported constantly interacting with black people, whether they were friends or family members, whereas 19% had little or no contact with this population.

Table 1 presents data regarding knowledge about ethnic-racial relations in health.

**Table 1:** Knowledge about ethnic-racial relations in health, São Paulo, São Paulo, Brazil (N=63)

	%			
	Agree	Disagree	Not sure	Did not answer
Racism is something that has been overcome in Brazil	-	-	100	-
Racism is a social determinant of health	90.5	7.9	1.6	-
There is a relationship between the race/color of a population and its health condition	80.9	12.7	4.8	1.6
The relationship between race/color and health condition is due exclusively to biological differences	4.8	7.9	87.3	-
The health condition of a population is not related to race/color, but rather to socioeconomic inequalities between social classes	39.7	15.9	44.4	-
The relationship between race/color and health condition is also a result of historical and social aspects	95.2	1.6	-	3.2

Table 2 presents information on knowledge about the black population’s health according to the year of graduation.

**Table 2:** Knowledge about the black population’s health by year, São Paulo, São Paulo, Brazil (N=63)

	n (%)					
	1^st^ year	2^nd^ year	3^rd^ year	4^th^ year	Graduates	Total
I do not know what the health issues are for the black population	2 (3.2)	1 (1.6)	-	1 (1.6)	-	**4 (6.3)**
I know little about the black population’s health	7 (11.1)	13 (20.6)	10 (15.9)	8 (12.7)	10 (15.9)	**48 (76.2)**
I am very familiar with the black population’s health issues	-	4 (6.3)	2 (3.2)	5 (7.9)	-	**11 (17.5)**
**Total**	**9 (14.3)**	**10 (28.6)**	**12 (19.0)**	**14 (22.2)**	**10 (15.9)**	**63 (100.0)**

When performing the Kruskal-Wallis test to compare the groups, no statistically significant difference was identified (X^2^=7.24; p=0.124).

**Figure 1:**
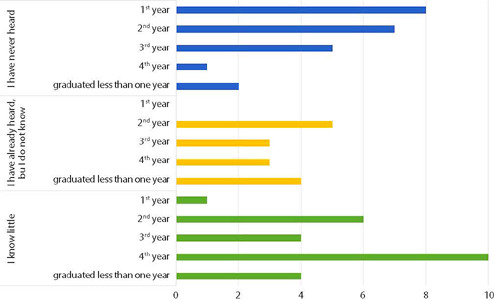
Knowledge regarding the Brazilian National Policy for Comprehensive Black Population’s Health by year, São Paulo, São Paulo, Brazil (N=63)

Participants have little knowledge about PNSIPN: 60.3% have never heard of it or do not know about it. Figure 1 shows the distribution between groups by year of graduation.

The comparison between groups demonstrated a statistically significant difference (X^2^=15; p=0.005). When the Dwass-Steel-Critchlow Fligner test was performed, it was identified that the difference occurred only between the 1^st^ and 4^th^ year groups (W=4,889; p=0.005).

The majority of participants (68.3%) indicated that the undergraduate nursing course was the main source of knowledge about health issues for the black population. However, only one third indicated the course as the main source of knowledge about PNSIPN. A total of 31 participants had never had access to PNSIPN (49.2%), among whom nine were in the 1^st^ year, nine in the 2^nd^ year, five in the 3^rd^ year, four in the 4^th^ year, and four were graduates.

There was a unanimous report that the black population’s health is addressed in the course, but 54% reported that it is rarely addressed, and 41.3% reported occasional attendance. According to participants, the black population’s health is addressed in the nursing course more frequently in relation to the historical and social factors of Brazilian society than in relation to its genetic and pathophysiological factors. The approach is carried out in the nursing course curricular units (60.3%) – with the majority of curricular units of the Department of Public Health mentioned –, university extension activities (38.1%), academic leagues (30.2%), curricular units of the basic cycle (27%), research (20.6%) and internship field (17.5%).

Thus, 60 participants (95.2%) agree that learning about PNSIPN during the nursing course is important and can bring about social transformations related to racism. Half of graduates disagree that their current knowledge about the policy is sufficient for their professional performance. Despite this, 70% agree that they would be able to provide assistance to this population, and 6.4% of students preferred not to respond.

## DISCUSSION

Racism is not just a set of individualized discriminatory actions, but a systemic process that should not be limited to isolated behavioral aspects^(9)^. All research participants agree that racism has not yet been overcome in Brazil. This data supports the findings of the research commissioned by the *Instituto de Referência Negra Peregum,* which identified that 81% of the Brazilian population onsiders Brazil to be totally or partially a racist country^(10)^. The recognition by 90.5% of research participants of racism as a social determinant of health is the first step towards combating institutional racism in the SUS and promoting racial equity in health, in accordance with PNSIPN assumptions and guidelines^(6)^. By agreeing that there is a relationship between a population’s race/color and its health condition, participants are better able to recognize the differences in the living and health conditions of communities and propose individual and collective care according to specific needs. However, the results of this research presented paradoxical data, since 39.7% believe that the health conditions of a population are not related to race/color, but rather to socioeconomic inequalities between social classes, and 15.9% are unsure about this issue. A study observed this same contradiction in a study carried out with 12 coordinators of different health courses in São Paulo. The coordinators interviewed considered that health is determined by “socio-environmental conditions”, and attributed the black population’s vulnerability to economic inequalities, not to racism^(11)^.

Disregard for racism in the processes of vulnerability is a reflection of the hegemonic belief in the myth of racial democracy, used to cover up the process of recognizing racial discrimination in an attempt to prevent and avoid racial conflict. It persists in the Brazilian imagination and aims to socialize black and white people as supposedly equal, generating a naturalization of racial inequities^(12, 13)^. Furthermore, the field of Brazilian public health was forged in a sociological tradition of privileging the category of social class to address social inequalities that consider the centrality of economic issues as determinants of health conditions^(14)^. Despite many advances in the production of knowledge and the formulation of public policies for the black population (driven by the movements), common sense still persists among students, educators and healthcare professionals.

The study demonstrated that students and recent graduates of the UNIFESP nursing course who participated in the research have little knowledge about the black population’s health and about PNSIPN. This result supports the findings in scientific literature. A study carried out in the context of Primary Care identified that nurses were unaware of PNSIPN and considered it unnecessary in the context of the SUS^(15)^. Another study found that the majority of residents and preceptors of Family Health and Family and Community Medicine Residency Programs were unaware of PNSIPN guidelines and objectives and the concept of institutional racism^(16)^. In both studies, participants (nurses, residents and preceptors) refer to the lack of formal and ongoing training in health for the black population during their undergraduate course and in the residency program.

Since no recent graduate responded that they were familiar with health issues related to the black population and the percentage of participants familiar with the topic does not increase as the course progresses, it is possible to infer that knowledge is not being acquired in the nursing course. This is supported by the data regarding the approach to the topic in the course, since the majority reported that the frequency of the approach is rare or occasional. Studies show that there is a systematic invisibility of ethnic-racial issues in health courses^(11, 17,18, 19)^. In medical courses, the approach to ethnic-racial issues emphasizes the healthcare dimension, directed towards genetic and biological issues with an emphasis on diseases prevalent in the black population^(17, 19)^. Unlike this finding, in the present study, it was observed that the black population’s health is more frequently correlated with historical and social factors of Brazilian society than with genetic and pathophysiological factors. Study participants mostly cited the Department of Public Health curricular units as those in which the topic is addressed.

Another study showed that the undergraduate course in public health has the highest percentage of credits for subjects that include the black population’s health^(19)^, demonstrating that the topic has greater insertion in public health. This study identified that, although equity is included conceptually in the guiding documents of a nursing course, the approach to the black population’s health depends on the initiative of specific professors^(19)^. Despite this, more than half of participants in this study had never heard of PNSIPN.Although the university is considered one of the main means of accessing the subject, it is only addressed sporadically during undergraduate studies, even though the Brazilian National Curricular Guidelines for Education on Ethnic-Racial Relations and for Teaching Afro-Brazilian and African History and Culture determine that higher education institutions must include the subject in their teaching plans^(20)^.

In addition to the Brazilian National Curricular Guidelines for Education on Ethnic-Racial Relations and for Teaching Afro-Brazilian and African History and Culture, PNSIPN itself provides for the inclusion of topics of racism and the black population’s health in health workers’ training processes^(6)^. A study analyzed the inclusion of the black population’s health in health courses from professors’ perspective and identified the following critical factors: lack of theoretical basis; traditional format of teaching-learning programs; lack of time; and productivity pressure from universities^(19)^. Higher education institutions need to reflect on their political culture, which is historically racist, elitist and hierarchical. When discussing the teaching-learning process on health issues for the black population, there is a certain resistance among professionals working in the health area to recognize and develop “awareness of privilege and disadvantage, racism and prejudice present in relationships”^(21)^.

The absence of ethnic-racial issues in the curricula of health courses is part of the tradition of Brazilian social thought of erasing the experiences and perspectives of those who are oppressed in a racialized society^(4)^. Public, private and civil society institutions, such as universities, transmit, through generations, a way of functioning that includes forms of exclusion and maintenance of privileges that are systematically denied and silenced. This process is conceptualized as “the whiteness pact”, defined by the unspoken complicity between white people who have narcissistic components, as it aims at self-preservation^(22)^. In this regard, it is important to highlight that only 23.3% of higher education teachers are black, with black women accounting for 10.6% and black men for 12.7%^(23)^. Whiteness is an ideological construct from which white people define themselves based on the denial of the humanity of other non-white population groups, originating from the process of colonization of territories by Europe. The idea of a “universal human being” was constructed from white racial identity, and from it derives the power structure that results in racial hierarchy, assigning social places to whites and non-whites^(24)^. Whiteness, as a system of power, attributes social privileges to the white population also in teaching institutions. Institutions are the materialization of a social structure or a mode of socialization that has coloniality and racism as organic components^(9)^. Thus, educational institutions are racist because society is racist. This does not mean accepting the facts, but indicating that, if universities wish to promote countercolonial changes, they will need to “act in a conflictual manner, positioning themselves within the conflict”^(9)^.

The lack of an approach to the topic of ethnic-racial relations in health education results in deficient professional performance and inability to offer responses that fill the gaps related to the specific health needs of the black population^(4)^. Health education centered on a body considered universal, the white body, contributes to training professionals who consider and practice a single way of caring and healing, and makes them insensitive to the specificities of other bodies, who hierarchize knowledge and practices. This type of incomplete academic training strengthens racism and health inequities, as these are professionals who are not qualified to listen to and live with other epistemes^(1)^.

The literature shows that initiatives to transform health education by including the topic of ethnic-racial relations in health courses have been carried out through optional/elective curricular units, not within the formal curriculum, upon notification by the Ministry of Education^(11, 25, 26)^. An experience report on implementing an optional course aimed at training healthcare professionals in combating racism, offered to students in health courses, recognized the optional course as powerful, as it promoted interdisciplinarity, interprofessionality and diversity through discussions in small groups about racism and its influences on health^(26)^. They identified that students from different courses in health did not understand the influence of racism in the clinical encounter, in anatomy classes and in the representation of the mannequins used to develop clinical practices^(26)^.

Nursing is made up of approximately 28 million professionals worldwide, and has a strong political role in changing social contexts and disseminating knowledge to society. Although nursing professionals act directly on health inequities and are key to changing perspectives on healthcare for vulnerable populations, there is a serious deficit of knowledge about the black population’s health^(27)^. There is an absence of the topic in curricula at all levels of education, including continuing education in healthcare services^(27)^. Nursing professionals have little or no knowledge about care practices inherited from African and indigenous cultures, which negatively impacts comprehensive healthcare promotion. There is a hegemony of biomedical thinking among nursing professionals, and the lack of knowledge and devaluation of Afro-indigenous health knowledge generates an asymmetry between professionals and users, in addition to therapeutic failure^(27)^. In this research, most of participants considered that their knowledge about PNSIPN is insufficient for their professional performance. It is essential that nursing professionals deepen their theoretical and practical knowledge regarding ethnic-racial relations in order to develop a critical and intersectional view of health conditions, understanding how social factors can act and influence health and quality of life.

### Study limitations

The main limitation of this study was that it was conducted in the nursing course of only one public university in southeastern Brazil. There may be discrepancies in other higher education institutions or other regions of the country. Moreover, the adoption of convenience sampling may generate a bias in the interpretation of results, since it is not representative of the universe.

### Contributions to nursing, health or public policy

Understanding nursing students’ and recent graduates’ perception regarding the issue of ethnic-racial relations in the black population’s health can contribute to including the topic in the debate on curricular updates for health courses, specifically nursing. As a field of knowledge and social and health practices, nursing can benefit from advances in knowledge production and, as a consequence, reverse poor health outcomes that affect the black population at different life cycles. Hence, promoting anti-racist education in nursing, capable of minimizing the chances of professionals reproducing racist practices in the context of healthcare and management, can reverse health inequities and qualify actions and strategies through health training processes.

Professional qualification and excellence in nursing education can only be achieved if the issue of racism and its relationship with health is included in training processes. This would enable the development of professional competence to address the specific needs of the black population, with the aim of achieving racial equity in health. This is an ethical requirement in order to achieve ideal levels of health provided for by law and guided by the SUS equity, comprehensiveness and universality principles.

## CONCLUSIONS

The majority of students and graduates of the UNIFESP nursing course who participated in this study have little knowledge about the black population’s health (76%), and 60.3% had never heard of PNSIPN. None of the recent graduates responded that they were familiar with the black population’s health issues, and there was no statistically significant difference between the percentages of participants familiar with the topic in the different groups by year of graduation. The vast majority reported that the frequency of addressing the topic in the nursing course is rare or occasional (90.5%).

The lack of knowledge on the subject may be related to the insufficient approach to the black population’s health during training. The fact that public health is the curricular unit with the greatest adherence to the subject reflects obstacles to the scope and mainstreaming of the subject in the nursing course. The controversy between the perception of low knowledge about the policy and the perception about the ability to work with the black population reveals the need for in-depth analysis of the possible factors that interfere with these contradictory perceptions.

The insufficient approach to the issue of the black population’s health in nursing courses corroborates the perpetuation of discriminatory and racist practices carried out daily by healthcare professionals, violating the SUS ethical principles. The inclusion and mainstreaming of the issue in nursing training requires efforts to deepen the debate on updating the curriculum. Filling this gap in training can contribute to qualifying the health workforce and, as a consequence, reverse the scenarios of worse health outcomes affecting the black population.

## Data Availability

https://osf.io/wju8a/?view_only=25dfaf5c8c2449a2a68d07 4285ca4d18
