# Supplementary material for: Black population’s health in nursing training: students’ knowledge
Source: Rev Bras Enferm. 2025 Jun 20;78(Suppl 1):e20240330. doi: 10.1590/0034-7167-2024-0330 (PMC12185114; doi:10.1590/0034-7167-2024-0330)
Supplement: Translation

# Translation: Saúde da população negra na formação em enfermagem: conhecimentos de estudantes

**DOI:** 10.1590/0034-7167-2024-0330pt

**Authors:** Gabriela Matos Rodrigues, Beatriz de Souza Lima, Eduardo Sodré de Souza, Hugo Fernandes, Suiane Costa Ferreira, Flávia Saraiva Leão Fernandes

**Affiliations:** I Universidade Federal de São Paulo. São Paulo, São Paulo, Brasil; II Faculdade Sírio-Libanês. São Paulo, São Paulo, Brasil; III Universidade Estadual de Campinas. Campinas, São Paulo, Brasil; IV Universidade do Estado da Bahia. Salvador, Bahia, Brasil

**Keywords:** Educação em Enfermagem, Ensino, Relações Raciais, Racismo, População Negra

## Abstract

**Objetivos:**

analisar o conhecimento de estudantes e recém-egressos/as de um curso de enfermagem sobre a saúde da população negra.

**Métodos:**

estudo exploratório e transversal, com estudantes e recém-egressos/as de enfermagem de uma universidade pública da região Sudeste do Brasil. Coletaram-se dados de forma *online* com 63 participantes. Realizou-se o teste de Kruskal-Wallis para análise estatística.

**Resultados:**

evidenciou-se baixo conhecimento sobre a saúde da população negra e sobre a Política Nacional da Saúde Integral da População Negra. A abordagem da temática no curso de enfermagem é rara e principalmente realizada em unidades curriculares da saúde coletiva, a partir de perspectiva sócio-histórica.

**Conclusões:**

o baixo conhecimento de estudantes revela a necessidade de maior abordagem da saúde da população negra no curso de enfermagem, com vistas à formação centrada nas relações étnico-raciais e práticas antirracistas.

## INTRODUÇÃO

As evidências científicas sobre a formação em saúde denunciam a grande lacuna que existe sobre o debate acerca do racismo e saúde da população negra, o que revela a urgência em repensar a formação em saúde, a fim de diminuir as iniquidades raciais e atender aos princípios da universalidade, integralidade e equidade no Sistema Único de Saúde (SUS)^(1)^.

Apesar de o SUS garantir legalmente o acesso universal e igualitário à saúde, as populações negras e indígenas enfrentam diariamente barreiras de acesso à saúde, refletidas nos seus desfechos de saúde, de doença e de morte. Essas barreiras são impostas pelo racismo institucional, que é evidenciado na análise comparada dos dados epidemiológicos desagregados segundo raça/cor, como precocidade de óbitos, altas taxas de mortalidade materna e infantil, maior prevalência de doenças infecciosas e crônicas, além de maiores índices de violência^(2, 3)^.

As evidências sobre atitudes negativas de profissionais de saúde durante a assistência à população negra refletem a dimensão interpessoal do racismo institucional nos serviços^(2)^. Tais atitudes, manifestadas por meio de imperícia, imprudência e negligência, contribuem para as iniquidades em saúde deste grupo, ao mesmo tempo que anunciam a necessidade de abordagem do tema nos cursos da área da saúde^(4)^.

A área da enfermagem tem posição privilegiada nos sistemas de saúde e, por isso, tem grande capacidade de produzir mudanças que contribuam para o desmantelamento do racismo institucional. Entretanto, o baixo nível de conhecimento e de estudos sobre a saúde desta população convoca para aprofundamentos neste campo. Portanto, a discussão sobre os impactos do racismo na determinação social da doença e atuação no enfrentamento dessas desigualdades deve ser incluída no processo de ensino-aprendizagem de futuros/as profissionais de enfermagem, a fim de promover equidade em saúde no Brasil e atender às diretrizes da Política Nacional de Saúde Integral da População Negra (PNSIPN)^(5, 6)^.

Fruto de intensa participação do movimento negro e de mulheres negras organizadas, a PNSIPN preconiza a produção de conteúdo, formação e educação permanente para trabalhadores/as de saúde. Tem como diretriz geral a “inclusão dos temas Racismo e Saúde da População Negra nos processos de formação e educação permanente dos trabalhadores da saúde e no exercício do controle social na saúde”^(6)^. Assim, destaca-se o papel das universidades públicas, pela sua capacidade de disseminar conhecimento e aumentar sua relevância nos meios de aprendizagem e formação acadêmica, tornando viável as transformações que podem ser alcançadas na reorganização da atenção à saúde por meio da adoção de ações institucionais globalizantes e includentes^(7)^.

Neste contexto, a Universidade Federal de São Paulo (UNIFESP) incorporou, nos seus documentos institucionais, diretrizes da PNSIPN. No seu Plano de Desenvolvimento Institucional 2021-2025^(8)^, propõe-se a se articular com as políticas para redução das desigualdades étnico-raciais, além de renovar projetos pedagógicos e práticas acadêmicas para o combate ao racismo e consolidar uma política institucional na temática. Em relação a esta última proposição, a universidade conta com a Política Carolina Maria de Jesus de Promoção da Equidade e Igualdade Étnico-Racial, Prevenção e Combate ao Racismo, que tem como um de seus eixos a “formação inicial e continuada em educação para as relações étnico-raciais”.

Diante do compromisso institucional assumido pela UNIFESP e da importância da formação de futuros profissionais para o combate ao racismo institucional no SUS, defende-se que a análise do conhecimento sobre a saúde da população negra oferecida em cursos de enfermagem pode contribuir para identificação e preenchimento de lacunas existentes. Desta maneira, este estudo teve o seguinte questionamento: qual o conhecimento de estudantes de enfermagem a respeito da saúde da população negra?

## OBJETIVOS

Analisar o conhecimento de estudantes e recém-egressos/as de um curso de enfermagem sobre a saúde da população negra.

## MÉTODOS

### Aspectos éticos

A pesquisa atendeu às normas da Resolução nº 466/2012, e foi submetida à apreciação do Comitê de Ética em Pesquisa da UNIFESP. Também houve aprovação da comissão do curso de enfermagem. O Termo de Consentimento Livre e Esclarecido (TCLE) foi submetido *online,* e recebeu a anuência das pessoas que aceitaram participar voluntariamente da pesquisa.

### Desenho, período e local do estudo

Trata-se de pesquisa exploratório-descritiva com delineamento transversal, norteada pela ferramenta *STrengthening the Reporting of OBservational studies in Epidemiology* (STROBE). Os dados foram coletados entre março e abril de 2023 na Escola Paulista de Enfermagem da UNIFESP. O curso de bacharelado em enfermagem é de regime integral anual, com tempo de integralização de quatro anos.

### População ou amostra; critérios de inclusão e exclusão

A população do estudo foi composta por discentes e recém-egressos/as de enfermagem da UNIFESP. A amostra foi obtida de forma não probabilística, por conveniência, a partir do envio de convite *online* para participação da pesquisa a todas as pessoas matriculadas nos quatro anos do curso de enfermagem em março de 2023 (288) e a todas as pessoas que colaram grau no final de 2022 (79), totalizando 367 pessoas.

Os critérios de inclusão foram estar matriculado/a no curso de enfermagem ou ter se formado no curso há menos de um ano no momento da coleta. Os critérios de exclusão foram estar com a matrícula trancada no momento da coleta ou demorar mais de 60 dias para resposta do instrumento. A amostra foi constituída por 63 participantes, que corresponde a 17,2% do total de pessoas convidadas a participar da pesquisa.

### Protocolo do estudo

O convite de participação da pesquisa com o *link* para o questionário virtual autoaplicável foi divulgado via secretaria acadêmica do curso para os endereços eletrônicos dos quatro anos do curso de graduação e da turma de egressos/as do ano anterior ao período de coleta de dados. O questionário virtual foi estruturado no sistema *Research Electronic Data Capture* (REDCap), contendo o TCLE e o instrumento de pesquisa elaborado pela equipe da pesquisa, para uso exclusivo neste estudo. O instrumento foi composto por perguntas fechadas e duas perguntas abertas, distribuídas em três partes: 1 - perfil sociocultural; 2 - conhecimentos sobre a saúde da população negra e a PNSIPN; e 3 - abordagem da temática no curso de enfermagem. Para analisar o conhecimento, foram elaboradas questões com respostas em escala tipo Likert de 5 pontos (1 - discordo totalmente a 5 - concordo totalmente), sendo possível analisar o nível de concordância diante de afirmações sobre a saúde da população negra.

Foi realizado teste piloto com um grupo de cinco estudantes pelo mesmo método que seria utilizado com os/as demais participantes. O objetivo era avaliar a compreensibilidade, objetividade, compreensão, formato, facilidade de preenchimento, entre outros. As alterações foram baseadas nas devolutivas dos/as estudantes. As respostas do teste piloto não foram incluídas na amostra final. A coleta de dados foi realizada após a aprovação do projeto no Comitê de Ética em Pesquisa e adaptações provenientes do teste piloto.

### Análise dos resultados e estatística

Utilizou-se o *software* Jamovi para análise dos dados. Na análise descritiva do conhecimento sobre a saúde da população negra, foram calculadas frequências absolutas e relativas. Na análise de associação, foi utilizado o ano da graduação como principal variável. Para a comparação entre os grupos 2º, 3º, 4º anos e recém-egresso/a), empregou-se o teste de Kruskal-Wallis, seguido do teste *Post Hoc Dwass-Steel-Critchlow-Fligner,* que compara a mediana/médias de todos os pares de grupos e controla a taxa de erro simultaneamente para todos os contrastes. Foi considerado o nível de significância de 0,05 para a interpretação dos resultados.

## RESULTADOS

Participaram do estudo 63 pessoas, sendo nove do 1º ano (14,3%), 18, do 2º ano (28,6%), 12, do 3° ano (19%), 14, do 4° ano (22,2%), e dez recém-egressos/as (15,9%). A amostra foi predominantemente composta por mulheres cisgêneras (90,5%), heterossexuais (61,9%), sem deficiência (96,8%), provenientes da região Sudeste do país (92%), pertencentes à faixa etária de 20 a 25 anos (69,8%), cuja média foi de 25 anos ± 6,65. Em relação ao quesito raça/cor, 68,3% se autoidentificaram como brancas, e 23,8% se autoidentificaram como negras, sendo 12,7% pardas e 11,1% pretas. A menor parte foi composta por pessoal amarelas (7,9%), e não houve participantes que se autodeclaram indígenas.

A maioria residia no município de São Paulo (87,3%), com os pais (66,7%) e não participava de nenhum programa de transferência de renda governamental (84,1%), com renda média entre três e cinco salários mínimos (42,9%). Em relação à escolaridade, 50,8% cursaram o ensino médio em escola particular, e a maioria cursava a primeira graduação (82,5%). Cerca de 80,9% referiram conviver constantemente com pessoas negras, sendo estes amigos ou familiares, enquanto que 19% conviviam pouco ou não possuíam nenhum contato com essa população.

Os dados referentes ao conhecimento sobre as relações étnico-raciais na saúde são apresentados na Tabela 1.

**Tabela 1:** Conhecimentos sobre relações étnico-raciais na saúde, São Paulo, São Paulo, Brasil (N=63)

	%			
	Concordo	Não tenho certeza		Discordo	Não respondeu
O racismo é algo superado no Brasil	-	-		100	-
O racismo é um determinante social da saúde	90,5	7,9		1,6	-
Existe relação entre raça/cor de uma população e sua condição de saúde	80,9	12,7		4,8	1,6
A relação entre raça/cor e a condição de saúde é decorrente exclusivamente de diferenças biológicas	4,8	7,9		87,3	-
A condição de saúde de uma população não tem relação com raça/cor, mas sim com as desigualdades socioeconómicas entre as classes sociais	39,7	15,9		44,4	-
A relação entre raça/cor e condição de saúde é decorrente também de aspectos históricos e sociais	95,2	1,6		-	3,2

As informações sobre o conhecimento sobre a saúde da população negra, de acordo com o ano da graduação, são apresentadas na Tabela 2.

**Tabela 2:** Conhecimentos sobre saúde da população negra por ano, São Paulo, São Paulo, Brasil (N=63)

	n (%)					
	1º ano	2º ano	3º ano	4º ano	Egressos/as	Total
Não sei quais são as questões de saúde da população negra	2 (3,2)	1 (1,6)	-	1 (1,6)	-	**4 (6,3)**
Conheço pouco sobre a saúde da população negra	7 (11,1)	13 (20,6)	10 (15,9)	8 (12,7)	10 (15,9)	**48 (76,2)**
Estou muito familiarizado com as questões de saúde da população negra	-	4 (6,3)	2 (3,2)	5 (7,9)	-	**11 (17,5)**
**Total**	**9 (14,3)**	**10 (28,6)**	**12 (19,0)**	**14 (22,2)**	**10 (15,9)**	**63 (100,0)**

Ao realizar o teste de Kruskal-Wallis para a comparação entre os grupos, não foi identificada diferença estatisticamente significante (X^2^=7,24; p=0,124).

Os/as participantes possuem baixo conhecimento sobre a PNSIPN: 60,3% nunca ouviram falar ou não a conhece. A Figura 1 apresenta a distribuição entre os grupos por ano de graduação.

**Figura 1:** Conhecimentos em relação à Política Nacional de Saúde Integral da População Negra por ano, São Paulo, São Paulo, Brasil (N=63)

A comparação entre os grupos demonstrou diferença estatisticamente significante (X^2^=15; p=0,005). Ao ser realizado o teste de Dwass-Steel-Critchlow Fligner, identificou-se que a diferença se deu apenas entre o grupo da 1ª série e da 4ª série (W=4.889; p=0,005).

A maioria dos/as participantes (68,3%) indicou que o curso de graduação em enfermagem foi a principal fonte de conhecimento sobre questões da saúde da população negra. Porém, apenas um terço apontou o curso como principal fonte de conhecimento sobre a PNSIPN. Um total de 31 participantes nunca tiveram acesso à PNSIPN (49,2%), entre os quais nove estavam no 1° ano, nove, no 2° ano, cinco, no 3° ano, quatro, no 4° ano, e quatro eram egressos(as).

Foi unânime o relato de que a saúde da população negra é abordada no curso, porém 54% referiram que a frequência é rara, e 41,3% referiram frequência ocasional. Segundo os/as participantes, a saúde da população negra é abordada no curso de enfermagem mais frequentemente relacionada aos fatores históricos e sociais da sociedade brasileira do que em relação aos seus fatores genéticos e fisiopatológicos. A abordagem é realizada em unidades curriculares do curso de enfermagem (60,3%) – sendo mencionadas majoritariamente unidades curriculares do Departamento de Saúde Coletiva –, atividades de extensão universitária (38,1%), ligas acadêmicas (30,2%), unidades curriculares do ciclo básico (27%), pesquisa (20,6%) e campo de estágio (17,5%).

Assim, 60 participantes (95,2%) concordam que aprender sobre a PNSIPN durante o curso de enfermagem é importante e pode provocar transformações sociais relacionadas ao racismo. Metade dos/as egressos discorda que os atuais conhecimentos sobre a política são suficientes para sua atuação profissional. Apesar disso, 70% concordam que seriam capazes de realizar assistência a esta população, e 6,4% dos/das discentes preferiram não responder.

## DISCUSSÃO

O racismo não se constitui apenas de um conjunto de ações discriminatórias individualizadas, mas de um processo sistêmico que não deve ser limitado a aspectos comportamentais isolados^(9)^. Todos/as os/as participantes da pesquisa concordam que o racismo ainda não está superado no Brasil. Esse dado corrobora os achados da pesquisa encomendada pelo Instituto de Referência Negra Peregum, onde foi identificado que 81% da população brasileira considera totalmente ou em parte que o Brasil é um país racista^(10)^.

O reconhecimento por parte de 90,5% dos/as participantes da pesquisa do racismo como um determinante social da saúde é o primeiro passo para o combate ao racismo institucional do SUS e a promoção da equidade racial em saúde, em conformidade com os pressupostos e diretrizes da PNSIPN^(6)^. Ao concordarem que existe relação entre raça/cor de uma população e sua condição de saúde, os/as participantes têm mais condições de reconhecer as diferenças nas condições de vida e saúde das coletividades e propor uma atenção individual e coletiva de acordo com as necessidades específicas. Porém, os resultados da pesquisa apresentaram dados paradoxais, uma vez que 39,7% acreditam que as condições de saúde de uma população não têm relação com raça/cor, mas sim com as desigualdades socioeconómicas entre as classes sociais, e 15,9% não têm certeza em relação a essa questão. Estudo realizado com 12 coordenadores de diferentes cursos de saúde em São Paulo encontrou a mesma contradição. Os/as coordenadores/as entrevistados/as consideraram que a saúde é determinada por “condições socioambientais”, e atribuem a vulnerabilidade da população negra a desigualdades econômicas, não ao racismo^(11)^.

A desconsideração do racismo nos processos de vulnerabilização é reflexo da crença hegemônica no mito da democracia racial, utilizado para encobrir o processo de reconhecimento da discriminação racial na tentativa de impedir e evitar o conflito racial. Ele persiste no imaginário brasileiro e visa socializar pessoas negras e brancas como supostamente iguais, gerando uma naturalização das iniquidades raciais^(12, 13)^. Além disso, o campo da saúde coletiva brasileira foi forjado em uma tradição sociológica de privilegiar a categoria de classe social para tratar as desigualdades sociais que consideram a centralidade das questões econômicas como determinantes das condições de saúde^(14)^. Apesar de haver muitos avanços na produção de conhecimento e na formulação de políticas públicas para a população negra (impulsionados pelos movimentos), o senso comum ainda persiste entre os/as estudantes, educadores/as e profissionais de saúde.

O estudo demonstrou que estudantes e recém-egressos/as do curso de enfermagem da UNIFESP participantes da pesquisa apresentam baixo conhecimento sobre a saúde da população negra e sobre a PNSIPN. Este resultado corrobora os achados na literatura científica. Estudo realizado no contexto da Atenção Básica identificou que enfermeiros/as desconheciam a PNSIPN e a consideraram desnecessária no contexto do SUS^(15)^. Outro estudo identificou que a maioria de residentes e preceptores/as de Programas de Residência de Saúde da Família e Medicina de Família e Comunidade desconhecia as diretrizes e objetivos da PNSIPN e o conceito de racismo institucional^(16)^. Em ambos os estudos, as pessoas participantes (enfermeiros/as, residentes e preceptores/as) relatam a ausência de formação formal e permanente em saúde da população negra durante o curso de graduação e no programa de residência.

Devido ao fato de nenhum/a recém-egresso/a ter respondido que estava familiarizado/a com as questões da saúde da população negra e de a porcentagem de participantes familiarizados/as com o tema não aumentar conforme a progressão no curso, é possível inferir que os conhecimentos não estão sendo adquiridos no curso de enfermagem. Isso é corroborado pelos dados referentes à abordagem no tema no curso, visto que a maioria referiu que a frequência da abordagem é rara ou ocasional. Estudos demonstram que há uma invisibilidade sistemática da temática étnico-racial nos cursos da área da saúde^(11, 17, 18, 19)^. Nos cursos de medicina, a abordagem da temática étnico racial tem ênfase na dimensão assistencial, direcionada para questões genéticas e biológicas com ênfase nas doenças prevalentes na população negra^(17, 19)^. Diferentemente desse achado, no presente estudo, foi observado que a saúde da população negra é mais frequentemente correlacionada a fatores históricos e sociais da sociedade brasileira do que com fatores genéticos e fisiopatológicos. Os/as participantes deste estudo citaram majoritariamente as unidades curriculares do Departamento de Saúde Coletiva como aquelas em que ocorre a abordagem da temática.

Outra pesquisa demonstrou que o curso de graduação em saúde coletiva possui o maior percentual de créditos de disciplinas que inserem a saúde da população negra^(19)^, demonstrando que a temática tem maior inserção no campo da saúde coletiva. Apesar disso, mais da metade dos participantes deste estudo nunca ouviu falar da PNSIPN. Esta pesquisa identificou que, apesar de a equidade estar inserida de forma conceitual nos documentos orientadores de um curso de enfermagem, a abordagem da saúde da população negra depende da iniciativa de professores/asespecíficos/as^(19)^. Apesar disso, mais da metade dos participantes deste estudo nunca ouviu falar da PNSIPN.

Ainda que a universidade seja apontada como um dos principais meios de acesso à temática, essa é tratada de forma esporádica durante a graduação, mesmo que as Diretrizes Curriculares Nacionais para a Educação das Relações Étnico-Raciais e para o Ensino de História e Cultura Afro-Brasileira e Africana determinem que instituições de ensino superior devem inserir o tema em seus planos de ensino^(20)^.

Além das Diretrizes Curriculares Nacionais para a Educação das Relações Étnico-Raciais e para o Ensino de História e Cultura Afro-Brasileira e Africana, a própria PNSIPN prevê a inclusão dos temas racismo e saúde da população negra nos processos de formação de trabalhadores/as da saúde^(6)^. Um estudo que analisou a inserção da saúde da população negra em cursos da área da saúde na perspectiva de docentes identificou os seguintes fatores críticos: falta de embasamento teórico; formato tradicional dos programas de ensino-aprendizagem; indisponibilidade de tempo; e pressão produtivista das universidades^(19)^. As instituições universitárias necessitam refletir sobre sua cultura política que é historicamente racista, elitizada e hierarquizada. Quando se discute o processo de ensino-aprendizagem sobre questões da saúde da população negra, nota-se certa resistência dos profissionais atuantes na área da saúde em reconhecer e desenvolver a “consciência do privilégio e desvantagem, racismo e preconceito presentes nas relações”^(21)^.

A ausência das questões étnico-raciais nos currículos dos cursos da área da saúde faz parte da tradição do pensamento social brasileiro de apagamento das vivências e perspectivas de quem é oprimido em uma sociedade racializada^(4)^. As instituições públicas, privadas e da sociedade civil, como a universidade, transmitem, através de gerações, um modo de funcionamento que inclui formas de exclusão e de manutenção de privilégios sistematicamente negados e silenciados. Esse processo é conceituado como o “pacto da branquitude”, definido pela cumplicidade não verbalizada entre pessoas brancas que possuem componentes narcísicos, pois visa à autopreservação^(22)^. Nesse sentido, é importante ressaltar que apenas 23,3% do magistério superior é composto por docentes negros/as, onde mulheres negras são 10,6% e homens negros são 12,7%^(23)^. A branquitude é um construto ideológico a partir do qual o branco se autodefine a partir da negação da humanidade de outros grupos populacionais não brancos, oriundo do processo de colonização de territórios pela Europa. A ideia do “ser humano universal” foi construída a partir da identidade racial branca, e dela deriva a estrutura de poder que resulta na hierarquização racial, atribuindo lugares sociais para os brancos e não brancos^(24)^. A branquitude, com um sistema de poder, atribui privilégios sociais à população branca também nos processos de ensino-aprendizagem no contexto das instituições de ensino. As instituições são a materialização de uma estrutura social ou de um modo de socialização que tem a colonialidade e o racismo como componentes orgânicos^(9)^. Assim, instituições de ensino são racistas porque a sociedade é racista. Isso não significa aceitar os fatos, mas indicar que, se as universidades desejam promover mudanças contracoloniais, precisarão “atuar de maneira conflituosa, posicionando-se dentro do conflito”^(9)^.

A ausência da abordagem do tema das relações étnico-raciais na formação em saúde resulta em atuação profissional deficiente e incapaz de oferecer respostas que preencham as lacunas relacionadas às necessidades em saúde específicas da população negra^(4)^. O ensino em saúde centrado em um corpo considerado universal, o corpo branco, contribui na formação de profissionais que consideram e praticam um único modo de cuidar e curar, e faz com que sejam insensíveis às especificidades de outros corpos, que hierarquizem saberes e práticas. Esse tipo de formação acadêmica incompleta fortalece o racismo e as iniquidades em saúde, pois são profissionais que não estão capacitados para ouvir e conviver com outras epistemes^(1)^.

A literatura demonstra que as iniciativas de transformar a formação em saúde com a inclusão da temática das relações étnico-raciais nos cursos de saúde vêm sendo realizadas por meio de unidades curriculares optativas/eletivas, não dentro do currículo formal, mediante a notificação do Ministério da Educação^(11, 25, 26)^. Um relato de experiência da implementação de uma disciplina optativa destinada à formação de profissionais de saúde no combate ao racismo, oferecida para estudantes de cursos de saúde, reconheceu a disciplina optativa como potente, pois promoveu a interdisciplinaridade, a interprofissionalidade e a diversidade por meio da discussão em pequenos grupos sobre o racismo e suas influências na saúde^(26)^. Os/as discentes de diferentes cursos da área da saúde não possuíam a compreensão da influência do racismo no encontro clínico, nas aulas de anatomia e na representatividade dos manequins usados para o desenvolvimento de práticas clínicas^(26)^.

A enfermagem é constituída por aproximadamente 28 milhões de profissionais no mundo, e possui grande participação política para mudança de contextos sociais e divulgação de conhecimento com a sociedade. Apesar da enfermagem atuar diretamente sobre as iniquidades em saúde e ser chave para a mudança de perspectivas do cuidado em saúde de populações vulnerabilizadas, há um grave déficit de conhecimento sobre a saúde da população negra^(27)^. Há uma ausência da temática nos currículos em todos os níveis de educação, incluindo a educação permanente nos serviços de saúde^(27)^. A enfermagem possui baixo ou nenhum conhecimento sobre as práticas de cuidado herdadas das culturas africana e indígena, intervindo negativamente na promoção do cuidado integral em saúde. Há entre profissionais de enfermagem uma hegemonia do pensamento biomédico, e o desconhecimento e a desvalorização dos saberes de saúde afro-indígenas geram uma assimetria entre profissional e usuários/as, além do insucesso terapêutico^(28)^. Na presente pesquisa, a maioria das pessoas participantes considera que seus conhecimentos sobre a PNSIPN são insuficientes para sua atuação profissional. É fundamental o aprofundamento teórico e prático dos/das profissionais de enfermagem quanto às relações étnico-raciais, a fim de desenvolver um olhar crítico e interseccional sobre as condições de saúde, entendendo como os fatores sociais poderão atuar e influenciar a saúde e qualidade de vida.

### Limitações do estudo

A principal limitação do estudo foi ter sido realizado no curso de enfermagem de apenas uma universidade pública da região Sudeste do Brasil. Pode haver divergências em outras instituições de ensino superior ou outras regiões do país. Além disso, a adoção da amostragem por conveniência pode gerar um viés de interpretação dos resultados, tendo em vista não ser representativa do universo.

### Contribuições para as áreas da enfermagem, saúde ou políticas públicas

Conhecer a percepção sobre o conhecimento de estudantes de enfermagem e recém-formados/as sobre a temática das relações étnico raciais na saúde da população negra pode contribuir para a inserção do tema no debate sobre a atualização curricular dos cursos da área da saúde, especificamente o de enfermagem. Como campo de conhecimentos e práticas sociais e da saúde, a enfermagem pode se beneficiar com avanços em relação à produção de conhecimentos e, como consequência, reverter quadros de piores resultados em saúde que recaem sobre a população negra em diferentes ciclos de vida. Nesse sentido, a promoção de uma educação antirracista em enfermagem, capaz de minimizar as chances de os/as profissionais reproduzirem práticas racistas no contexto da assistência e gestão em saúde, pode reverter iniquidades em saúde e qualificar as ações e estratégias por meio de processos formativos em saúde.

A qualificação profissional e a excelência no ensino de enfermagem só poderão ser alcançadas à medida que se inclua a temática do racismo e sua relação com a saúde nos processos de formação. Possibilitaria o desenvolvimento de competência profissional para abordar as necessidades específicas da população negra, com o intuito de atingir a equidade racial em saúde. Trata-se de uma exigência ética no sentido de alcançar níveis ideais de saúde previstos em leis e orientados pelos princípios de equidade, integralidade e universalidade do SUS.

## CONCLUSÕES

A maioria de estudantes e egressos/as do curso de enfermagem da UNIFESP participantes deste estudo possui baixo conhecimento sobre a saúde da população negra (76%), e 60,3% nunca ouviram falar da PNSIPN. Nenhuma das pessoas recém-egressas respondeu que estava familiarizada com as questões da saúde da população negra, e não houve diferença estaticamente significativa entre as porcentagens de participantes familiarizados/as com o tema nos diferentes grupos por ano de graduação. A grande maioria referiu que a frequência da abordagem da temática no curso de enfermagem é rara ou ocasional (90,5%).

O baixo conhecimento sobre a temática pode estar relacionado à abordagem insuficiente da saúde da população negra durante a formação. O fato de a saúde coletiva ser a unidade curricular com maior aderência à temática reflete obstáculos ao alcance e à transversalização do tema no curso de enfermagem. A controvérsia entre a percepção de baixo conhecimento sobre a política e percepção sobre a capacidade de atuação com a população negra revela a necessidade de aprofundamentos sobre os possíveis fatores que interferem nessas percepções contraditórias.

A abordagem insuficiente do tema da saúde da população negra nos cursos de enfermagem corrobora a perpetuação de práticas discriminatórias e racistas operacionalizadas cotidianamente por profissionais de saúde, ferindo os princípios éticos do SUS. A inclusão e a transversalização do tema na formação em enfermagem exigem esforços para o adensamento do debate sobre atualização do currículo. Preencher essa lacuna na formação pode contribuir para qualificar a força de trabalho em saúde e, como consequência, reverter os quadros de piores resultados em saúde que incidem sobre a população negra.

## Data Availability

https://osf.io/wju8a/?view_only=25dfaf5c8c2449a2a68d07 4285ca4d18
